# You are not always what you eat—Fatty acid bioconversion and lipid homeostasis in the larvae of the sand mason worm *Lanice conchilega*

**DOI:** 10.1371/journal.pone.0218015

**Published:** 2019-06-06

**Authors:** Rita M. Franco-Santos, Holger Auel, Maarten Boersma, Marleen De Troch, Martin Graeve, Cedric L. Meunier, Barbara Niehoff

**Affiliations:** 1 Marine Zoology, University of Bremen, Bremen, Germany; 2 Polar Biological Oceanography, Alfred-Wegener-Institut Helmholtz-Zentrum für Polar- und Meeresforschung, Bremerhaven, Germany; 3 Marine Biology, Ghent University, Gent, Belgium; 4 Biologische Anstalt Helgoland, Alfred-Wegener-Institut Helmholtz-Zentrum für Polar- und Meeresforschung, Helgoland, Germany; 5 Ecological Chemistry, Alfred-Wegener-Institut Helmholtz-Zentrum für Polar- und Meeresforschung, Bremerhaven, Germany; MARE – Marine and Environmental Sciences Centre, PORTUGAL

## Abstract

The meroplanktonic larvae of benthic organisms are an important seasonal component of the zooplankton in temperate coastal waters. The larvae of the reef-building polychaete *Lanice conchilega* contribute up to 15% of the summer zooplankton biomass in the North Sea. Despite their importance for reef maintenance (which positively affects the benthic community), little is known about the trophic ecology of this meroplanktonic larva. Qualitative and quantitative estimates of carbon (C) transfer between trophic levels and of fatty acid (FA)—specific assimilation, biosynthesis, and bioconversion can be obtained by compound-specific stable isotope analysis of FA. The present work tested the hypothesis that the concept of fatty acid trophic markers (FATM), widely used for studies on holoplankton with intermediate to high lipid contents, is also applicable to lipid-poor organisms such as meroplanktonic larvae. The incorporation of isotopically-enriched dietary C by *L*. *conchilega* larvae was traced, and lipid assimilation did not follow FA-specific relative availabilities in the diet. Furthermore, FAs that were unavailable in the diet, such as 22:5(n-3), were recorded in *L*. *conchilega*, suggesting their bioconversion by the larvae. The results indicate that *L*. *conchilega* larvae preferentially assimilate certain FAs and regulate their FA composition (lipid homeostasis) independently of that of their diet. Their quasi-homeostatic response to dietary FA availability could imply that the concept of FATM has limited application in lipid-poor organisms such as *L*. *conchilega* larvae.

## Introduction

The meroplanktonic larvae of macrobenthic organisms are an important seasonal component of the zooplankton in temperate coastal waters [1]. In the North Sea, the larvae of the sand mason worm *Lanice conchilega* (Pallas, 1766) contribute up to 15% of the zooplankton biomass between July and September [2], when water temperatures are above 13°C. The reproductive peak of *L*. *conchilega* in the southern North Sea occurs in spring and is followed by smaller peaks during the summer and autumn [3], though larval supply can vary between years [4]. The larvae of this polychaete evolve from a short planktonic to a benthic phase and, after a few days, to a second planktonic stage, the aulophore larvae [5]. At this stage larvae already present a (transparent) tube, and display morphological features of a juvenile [6]. They are able to feed in the water column, where they can remain up to 60 days before succeeding to a benthic stage [7]. The benthic, tube-dwelling adults of *L*. *conchilega* are non-selective suspension-deposit feeders [8–10], which derive their diet from the organic matter available in the water column and in the sediment. Common food items include diatoms, bacteria and microphytobenthos, and the species is able to switch between food sources [8,10,11].

*Lanice conchilega* forms reefs capable of structuring the surrounding habitat and creating a complex and heterogeneous environment [12,13]. The reefs provide refuge against predation and physical and chemical stresses, attract a variety of other organisms, and serve as nursery and feeding grounds to several species, including fish and birds [14–19]. *Lanice conchilega* is an ecosystem engineer whose presence favors species richness and faunal abundance and positively affects the benthic community [12,13,19,20].

In comparison with adult organisms, few studies have been conducted on the ecology of early stages of *L*. *conchilega*. These mostly address topics such as juvenile settlement and buoyancy [4,7,21,22], and little information is available on the feeding ecology of the larvae. The survival and development of these meroplanktonic larvae and, thus, the maintenance of *L*. *conchilega* reefs, depend, a.o., on their feeding success. To the best of our knowledge there are no studies published on the feeding ecology of the larvae, a gap which needs to be addressed if the larval stage of this species is to be better understood.

Information on the feeding ecology of an organism on short and long time scales, as well as on the pathways of energy flow between prey and predator, can be obtained from the analysis of fatty acid trophic markers (FATM) [23] in combination with the use of stable isotopes. Fatty acid trophic markers are usually assimilated in a conservative manner (i.e., largely unmodified) by consumers [24,25]. The FAs 16:1(n-7), 16:2(n-4), 16:3(n-4), and 20:5(n-3) (eicosapentaenoic acid, EPA) are characteristic of diatoms, whereas 18:4(n-3) and 22:6(n-3) (docosahexaenoic acid, DHA) are considered dinoflagellate markers [8,23,26,27]. Compound-specific stable isotope analysis (CSIA) is a powerful tool for tracing organic matter origin and fate and to investigate biochemical processes and patterns in individuals and ecosystems (e.g., [28–30]). It provides accurate and precise measurements of the isotopic composition of compounds (up to 0.3‰ or 0.0003 atom percent excess, APE) [31]. When applied together with FA determination, CSIA highlights minute changes in FA-specific concentration. When used in feeding experiments with enriched diets, CSIA enables the discrimination of the origin of a FA–internal or dietary–, and also provides quantitative estimates for the transfer of lipid dietary carbon (C) from prey to predator and for FA-specific assimilation and potential biosynthesis and bioconversion [26,27,32,33].

The present work had two objectives. The first was to start filling the gap of knowledge on the feeding ecology of *L*. *conchilega* larvae. Experiments investigating FA-related C assimilation have been conducted mostly for holoplanktonic organisms with intermediate to high lipid contents, whose FA profile tends to reflect that of the food items they ingest (e.g., [26,27]). Little is known, however, about how meroplanktonic organisms, which are usually lipid-poor (total lipid content ranging between 5–15% dry mass), incorporate dietary FAs. It is possible that the lipid content and, thus, requirement, of an organism can dictate whether it will assimilate dietary FAs in an unmodified manner or not. We assume that the larvae of *L*. *conchilega* are lipid-poor, so our second objective was to test the hypothesis that the concept of FATM can also be applied in a feeding study with lipid-poor meroplanktonic larvae. In order to do so, the polychaete larvae were fed with a diatom culture previously enriched with ^13^C and the incorporation of isotopically enriched FA-specific lipid C into the consumer was followed. This allowed for the recording of C assimilation and turnover in the organism, and also for the investigation of possible bioconversion pathways.

## Method

### Field sampling

Zooplankton samples for the feeding experiment were collected on June 6^th^ 2016 with a 500 μm mesh-size CalCOFI net, which was towed horizontally for 15 minutes at 5 m depth off the German island of Helgoland (54^o^11’N, 07^o^54’E), in the southern North Sea. As zooplankton samples are routinely collected at this location for the Helgoland Roads time series, no specific permissions were required for this location/sampling. Samples were taken to the laboratory and intact and active aulophore larvae of the polychaete *L*. *conchilega* were immediately sorted under an Olympus SZX16 stereoscopic microscope.

### Algae culture

Batch cultures were created daily for *Conticribra weissflogii* (Grunow) (Stachura-Suchoples and Williams, 2009) for five consecutive days by diluting a stock solution with fresh f/2 medium (after [34]). Diatoms were enriched with ^13^C by adding ^13^C-enriched sodium bicarbonate (NaH^13^CO_3_) to the medium at a concentration of 4 mg L^-1^. Cultures were kept in constant light inside a temperature-controlled room at 18°C, and stirred twice a day in order to keep cells suspended. Algae were grown for five days and then used as food suspension (exponential growth phase) for the polychaete larvae during the experiment. *Conticribra weissflogii* cultures were sampled daily (5 days) during the experiment for determination of cell C, nitrogen (N), and FA content and of FA-specific ^13^C isotopic enrichment, though only 4 of the 5 samples yielded good chromatograms for the FA analysis. This was done by filtering subsamples of known cell concentrations through pre-combusted (500°C for 24 h) Whatman GF/F filters (0.7 μm pore size, 25 mm diameter). Filters for determination of prey C and N content were dried at 60°C for 48 h, folded inside aluminum foil, and stored in a desiccator until analysis. Samples for FA analyses were placed into pre-combusted lipid vials and stored at -80°C. The remaining volume of the cultures was then used to feed polychaete larvae. Cell densities were determined with a BD Accuri C6 Flow Cytometer.

### Experimental design

A total of 390 larvae were sorted, 30 for determination of *in situ* body C and N content, 150 for analyzing the *in situ* FA content and composition, and 210 for the feeding experiment. The enriched *C*. *weissflogii* was used as food source in the feeding experiment with *L*. *conchilega* larvae. Feeding experiments with *L*. *conchilega* were initiated immediately after sorting of the larvae. Individuals were kept for five days in groups of 70 individuals in triplicate 500 mL glass beakers fitted with a 300 μm meshed-bottom cylinder (140 ind L^-1^). The diatom suspension was provided on a daily basis at a concentration of 8000 cells mL^-1^. The polychaete larvae were kept in a dark temperature-controlled room at the temperature recorded when they were sampled *in situ*, i.e., 13.5 ± 0.3°C. The beakers were gently stirred three times a day for food resuspension in the water. A partial water exchange (66%) was performed daily and followed by the addition of new algae culture, with a final diatom concentration > 8000 cells mL^-1^. At the end of the experiment the larvae were sampled from each replicate for body C and N content determination and for analysis of FA composition and FA-specific ^13^C isotopic enrichment (n = 3, with 10 and 50 individuals per sample for C/N and FA analyses, respectively). Individuals (inside their tubes) were gently washed in distilled water, placed into pre-weighed tin capsules (5*9 mm, IVA Analysentechnik) for body C and N content determination or into pre-combusted glass vials for FA analyses, and stored at -80°C until further analysis. Tin capsules with larvae were dried at 60°C for 48 h, weighed with an ultra micro-balance (detection limit: 0.1 μg; XP6U Ultra Micro Balance, Mettler Toledo, Germany) and stored in a desiccator until analysis.

### Carbon, nitrogen and fatty acid content analyses

The C and N contents of all larvae and algae samples were measured with an elemental analyzer (detection limit: 2 μg C / 0.5 μg N; maximum error: ± 3%, Euro EA 3000, EuroVector S.P.A., Milan, Italy) using acetanilide as a standard.

Lipids were extracted and FAs identified as described by Boissonnot et al., 2016 [26]. Samples were homogenized in a dichloromethane:methanol (2:1, v:v) solution, from which total lipids were extracted. A known amount of an internal standard, the tricosanoic acid methyl ester (23:0), was added to each sample. Potassium chloride (KCl 0.88% solution) was added to create a biphasic system and aid in extraction. Lipid extracts were transesterified by heating samples with 3% sulfuric acid (H_2_SO_4_) in methanol at 80°C under nitrogen atmosphere for 4 hours. Fatty acid methyl esters (FAMEs) were then extracted with cyclohexane, and determined and quantified with a gas chromatograph (HP 6890 N, Agilent Technologies Deutschland GmbH & Co. KG) equipped with a 60m × 0.25mm i.d. wall-coated open tubular capillary column (film thickness: 0.25 μm; liquid phase: DB-FFAP), a split/splitless injector (250°C) and a flame ionization detector (280°C). The peaks in the chromatograms generated were manually identified in relation to known standards analyzed together with samples. Chromatograms were then evaluated with the ChemStation software from Agilent. The A:B(n-X) shorthand notation was used to refer to FAs, where A is the number of carbon atoms, B is the number of double bonds, and (n-X) gives the position of the double bond closest to the terminal methyl group. Total lipid contents of larvae and algae cultures were calculated by adding the mass of all FAs. Fatty acid concentrations are presented in absolute (ng C ind^-1^) and relative (% of total FA, TFA) units in order to report on both the quantity of FAs available in the samples and the relative importance of each FA within the total FAs for each sample (respectively). Fatty alcohols were not detected in the samples.

### Compound-specific stable isotope analysis (CSIA)

The FA-specific stable isotope composition of carbon in FAMEs extracted was obtained according to Boissonnot et al., 2016 [26] with a Thermo gas chromatography-combustion-isotope ratio mass spectrometry (GC-c-IRMS) system, equipped with a Trace GC Ultra gas chromatograph, a GC Isolink, and a Delta V Plus isotope ratio mass spectrometer connected via a Conflo IV interface (Thermo Scientific Corporation, Bremen, Germany). The chromatograms containing peak areas and C isotope ratios were analyzed with the IRMS software Isodat 3.0. The 14:0 and 18:0 FAME reference standards (Iowa University) with known δ-values used for further calculations.

The equations used by Boissonnot et al., 2016 [26] to calculate carbon assimilation were also applied in the present study, and were:

Isotopic ratios of FAs

$$\delta^{13}C\;\left(‰\right)=\left[\left(\frac{R_{\mathrm{sample}}}{R_{\mathrm{standard}}}\right)-1\right]*\;1000,$$ (1)

where *R*_sample_ and *R*_standard_ are the ratio of ^13^C/^12^C in the sample and in the Vienna Pee De Belemnite reference standard, respectively;

Atom percent (atom%), which are converted δ-values and express isotope data in terms of isotope concentrations to inform on the ^13^C enrichment in each FA

$$atom\%=\left(\frac{R_{sample}}{{(R}_{sample}+1)}\right)*100,$$ (2)

where R is the ratio ^13^C/^12^C in the sample;

Atom percent excess (APE), which considers the isotopic enrichment in experimental larvae (in comparison to *in situ* individuals) due to the assimilation of algae enriched in ^13^C

$$APE={atom\%}_{experimental\;polychaetes}-{atom\%}_{in\;situ\;polychaetes}$$ (3)

Carbon mass of each FA (C_mass_, in μg C ind^-1^), which divides the FA mass by the FAME mass

$$C_{mass}=\frac{\{(atom\%/100*A_{13C})+[(100-atom\%)/100*A_{12C}]\}*C_{FA}*{FA}_{mass}}{\left\{[(atom\%/100*A_{13C})+((100-atom\%)/100*A_{12C})]*C_{FA}\}+A_{12C}+(H_{FAME}*A_{H})+(O_{FAME}*A_{O}\right)},$$ (4)

where A_12C_, A_13C_, A_H_, and A_O_ are the atomic masses of ^12^C, ^13^C, H and O, respectively, C_FA_ is the number of carbon atoms in the FA, H_FAME_ and O_FAME_ are the number of hydrogen and oxygen atoms in the FAME, and FA_mass_ is the mass (in μg ind^-1^) of the FA;

Proportion of carbon assimilated (PA)

$$PA=\frac{APE}{L},$$ (5)

where L is the average enrichment (in atom%) in all algae FAs but 18:0 (L = 3.62%). The averaging is to account for the elongation and/or desaturation of small amounts of dietary FAs when they are assimilated by the larvae [23], and the exclusion of 18:0 is due to its poor enrichment in comparison to other FAs in prey cultures [26,35]. When calculating the PA in the larvae FA 18:0, L was the average of enrichment in algae FA 18:0 (L_FA 18:0_ = 1.87%);

Carbon assimilation (C_assim_, in μg C ind^-1^), calculated for each FA

$$C_{assim}=C_{mass}*PA$$ (6)

Total C assimilation was obtained by summing the C_assim_ values for all FAs. Assimilation rates were obtained by dividing C_assim_ by the number of experimental days when sample was collected;

Carbon turnover rates (C_T_ in % day^-1^)

$$C_{T}=\frac{\frac{C_{assim(t)}}{{C_{mass}}_{\left(t\right)}}}{\Delta t},$$ (7)

where _(t)_ indicates the specific sampling time for which the values of C_assim_ and C_mass_ should be used and Δt the number of experimental days when sample was collected.

### Statistical analyses

Differences in atom% for the different batches of the diatom culture were investigated with one-way analysis of variance (ANOVA), and the origin of differences was identified by applying the Tukey HSD (Honestly Significant Difference) post-hoc test with a 95% confidence limit. Prior to the ANOVAs, the data were tested for normality and homogeneity of variances with Shapiro-Wilk and Bartlett tests, respectively. The difference between *in situ* and laboratory-fed polychaete larvae in dry mass (DM), C, N, and total lipid contents, and C:N molar ratio were investigated with a t-test (for each variable). Data used for t-tests was also previously tested for normality and homogeneity of variances. Whenever a *p-value* was obtained, significance was set at *p* ≤ 0.05. All univariate analyses were performed using R ver. 3.4.4 [36].

Multivariate analysis of FA composition for *L*. *conchilega* individuals was performed with a dendrogram, which clustered individuals with similar FA profiles (based on their group average linkage clustering). The dendrogram was generated from a Bray-Curtis similarity matrix obtained from absolute and percentage (with logit transformation, as suggested by Warton and Hui, 2011 [37]) data on FAs with relative content > 1% TFA. All multivariate analyses were performed with PRIMER 7.0 software [38].

## Results

### Algal composition and enrichment

**Fig 1:**
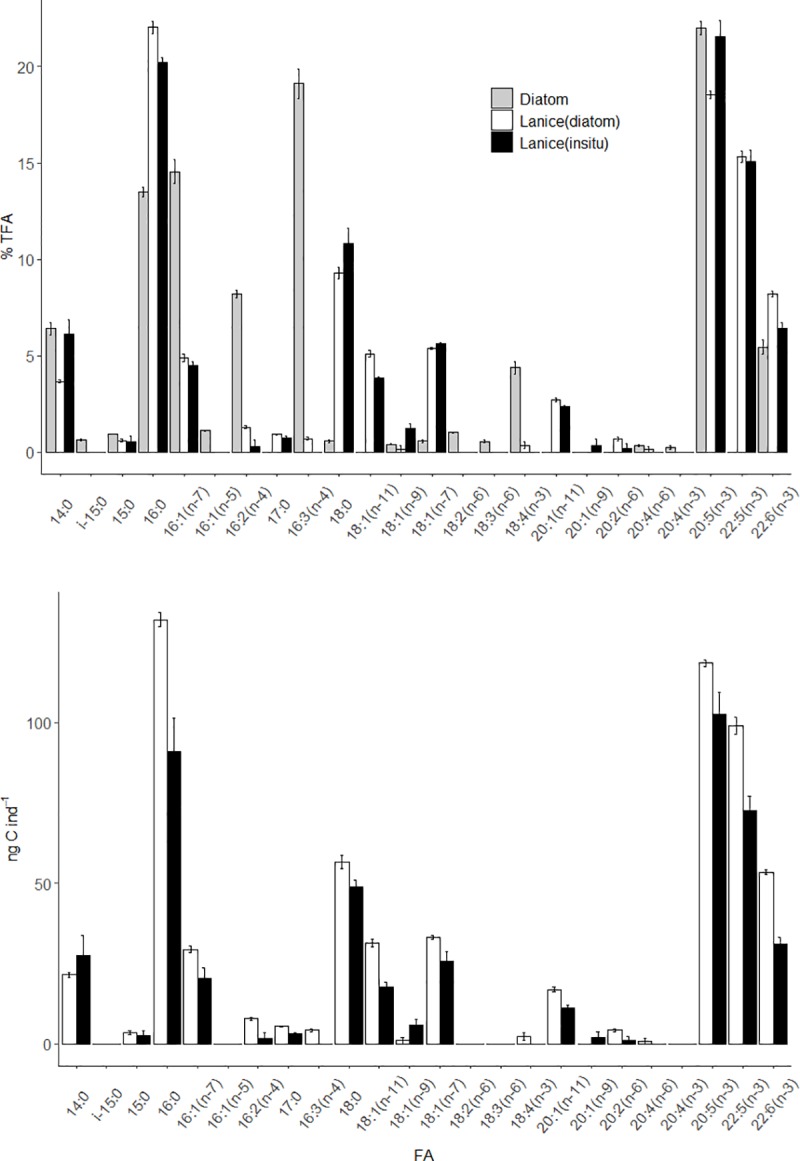
Fatty acid relative (% total FA, %TFA) and absolute (ng C ind^-1^)concentrations in diatoms (*C*. *weissflogii*, n = 4) and in *L*. *conchilega* larvae (n = 3 with 50 individuals pooled per sample). Data for larvae are differentiated between organisms sampled *in situ* and after 5 days of feeding on the diatom. Standard error bars are shown for the mean values.

The average C and N contents of the *C*. *weissflogii* cultures were 577 and 117 μg L^-1^, respectively, resulting in an average molar C:N ratio of 5.8 (Table 1). The average lipid content was 116 μg L^-1^ (Table 1). The FA profile of the diatom (Table 1, Fig 1) was dominated by polyunsaturated fatty acids (PUFAs), which comprised 61% of total fatty acids (TFA). Saturated (SFA) and monounsaturated (MUFA) fatty acids were present in similar amounts, 22 and 17% TFA, respectively. The most important FAs in the *C*. *weissflogii* batches were 20:5(n-3) (EPA), 16:3(n-4), 16:1(n-7), and 16:0 (22, 19, 15 and 13% TFA, respectively). The FA 22:6(n-3) (DHA) was present in a smaller amount, i.e., 5% of TFA.

**Table 1:** Dry mass (DM, in μg ind^-1^); carbon (C), nitrogen (N) and total lipid contents (TLC) (ng larvae^-1^ / diatom: μg L^-1^ (pg cell^-1^)); molar C:N ratio; and FA-specific absolute (ng C larvae^-1^) and relative (larvae: ‰ body C (italics, second row) / diatom: ‰ cell C / all: % total FA, in parenthesis) composition of *L*. *conchilega* larvae and its diet, the diatom *C*. *weissflogii*.

	*L*. *conchilega*			*C*. *weissflogii*
	*in situ*	Experiment		
		Internal and dietary C	Internal C	
DM	55 ± 6	52 ± 13		
C	7668 ± 2858	8511 ± 966		577 ± 44 (72 ± 6)
N	1929 ± 628	2170 ± 270		117 ± 11 (15 ± 1)
Molar C:N ratio	4.6 ± 0.3	4.6 ± 0.1		5.8 ± 0.5
TLC	632 ± 116	845 ± 11		116 ± 13 (15 ± 2)
ΣFAs	465 ± 85 *66 ±* 25	622 ± 8 *74 ± 10*	423 ± 26	148 ± 18
14:0	28 ± 11 (6 ± 1) *4 ± 1*	21 ± 1 (4 ± 0) *3 ± 0*	16 ± 2	9 ± 2 (6 ± 1)
16:0	91 ± 18 (20 ± 0) *13 ± 5*	132 ± 4 (22 ± 1) *16 ± 2*	88 ± 8	19 ± 3 (13 ± 0)
16:1(n-7)	20 ± 5 (4 ± 0) *3 ± 1*	29 ± 2 (5 ± 0) *3 ± 0*	16 ± 0	21 ± 4 (15 ± 1)
16:1(n-5)	N.D.	N.D.	N.D.	2 ± 0 (1 ± 0)
16:2(n-4)	N.D.	8 ± 1 (1.3 ± 0) *1 ± 0*	4 ± 0	12 ± 2 (8 ± 0)
16:3(n-4)	N.D.	4 ± 1 (0.7 ± 0) *1 ± 0*	2 ± 0	28 ± 3 (19 ± 2)
18:0	49 ± 4 (11 ± 1) *7 ± 4*	57 ± 3 (9 ± 1) *7 ± 1*	37 ± 6	1 ± 0 (1 ± 0)
18:1(n-11)	18 ± 3 (4 ± 0) *3 ± 1*	31 ± 2 (5 ± 0) *4 ± 1*	26 ± 3	N.D.
18:1(n-9)	6 ± 3 (1.2 ± 0) *1 ± 0*	N.D. N.D.	1 ± 2	1 ± 0 (0.4 ± 0)
18:1(n-7)	26 ± 5 (6 ± 0) *4 ± 1*	33 ± 1 (5 ± 0) *4 ± 1*	24 ± 1	1 ± 0 (1 ± 0)
18:2(n-6)	N.D.	N.D.	N.D.	1 ± 0 (1 ± 0)
18:4(n-3)	N.D.	3 ± 0 (0.5 ± 0)	1 ± 1	7 ± 2 (4 ± 1)
20:1(n-11)	11 ± 2 (2 ± 0) *2 ± 1*	17 ± 1 (3 ± 0) *2 ± 0*	15 ± 2	N.D.
20:5(n-3)–EPA	103 ± 12 (22 ± 1) *15 ± 6*	119 ± 2 (19 ± 0) *14 ± 2*	77 ± 3	34 ± 3 (22 ± 1)
22:5(n-3)	73 ± 8 (15 ± 1) *10 ± 4*	99 ± 5 (15 ± 1) *12 ± 2*	74 ± 6	N.D.
22:6(n-3)–DHA	31 ± 4 (6 ± 0) *4 ± 2*	53 ± 1 (8 ± 0) *6 ± 1*	32 ± 1	8 ± 1 (5 ± 1)
ΣSFA	173 ± 34 (38 ± 1) *25 ± 10*	219 ± 8 (36 ± 1) *26 ± 4*	148 ± 15	32 ± 5 (22 ± 1)
ΣMUFA	83 ± 22 (18 ± 1) *12 ± 4*	112 ± 1 (18 ± 0) *13 ± 2*	81 ± 5	24 ± 5 (17 ± 1)
ΣPUFA	209 ± 29 (44 ± 2) *30 ± 11*	291 ± 8 (45 ± 1) *34 ± 4*	194 ± 8	92 ± 9 (61 ± 2)

Total lipid C content is given by the ΣFAs term. A distinction is made between the internal (non-enriched, from *in situ* origin) and the internal + dietary (enriched, assimilated from the diet) FA contents in *L*. *conchilega* at the end of the feeding experiment. Only FAs > 1% TFA are indicated. Values are means ± SD from samples and from the sum of saturated (SFA), monounsaturated (MUFA) and polyunsaturated (PUFA) fatty acids (n = 3 for larvae (50 individuals pooled per sample) and n = 4 (for FA values) or 5 (for C and N values) for diatoms). N.D.: not detected.

**Fig 2:**
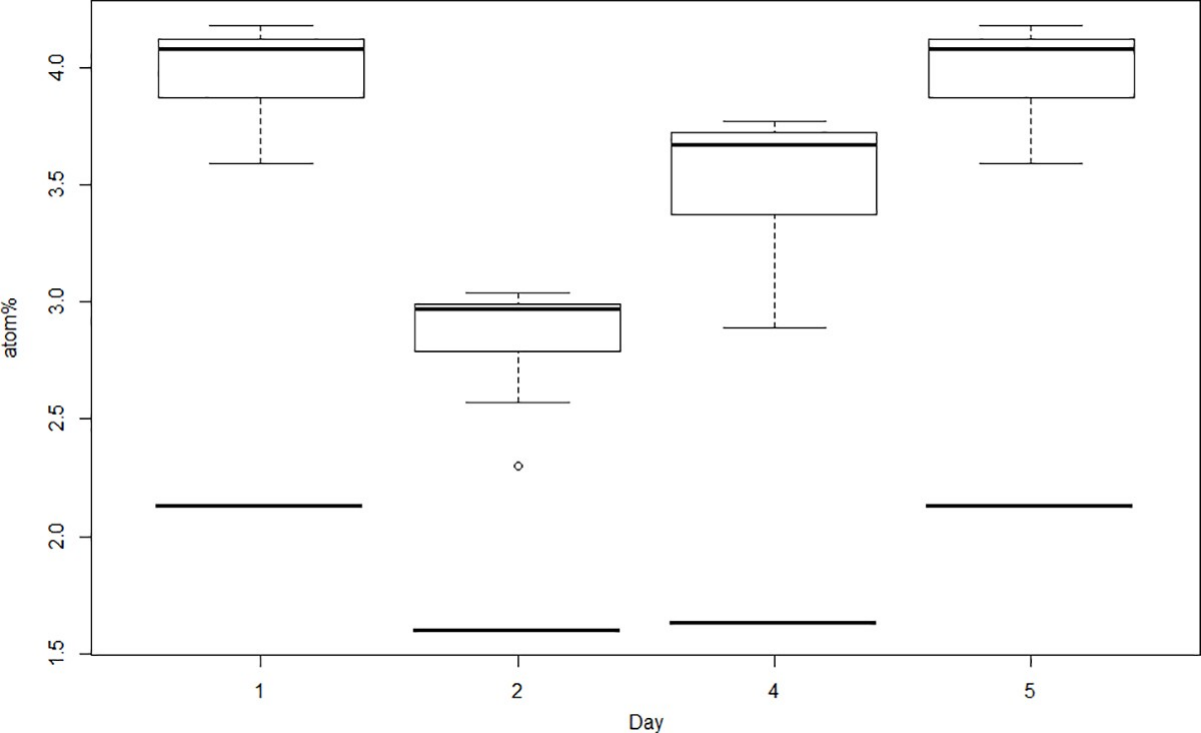
^13^C isotopic enrichment (atom%) of *C*. *weissflogii* daily batch cultures fed to polychaete larvae during the experiment. Straight lines represent atom% values for the fatty acid (FA) 18:0, whereas boxplots and open circles represent average atom% values from all the other FAs. The minimum and maximum observation values are represented by the lower and upper whiskers (respectively), the first and third quartiles are represented by the lower and upper hinges (respectively), and the median is shown as the line that divides the box.

The ^13^C isotopic enrichment in all diatom FAs (but 18:0) varied from 2.88 ± 0.21 atom% to 4.00 ± 0.19 atom% between the different batches, averaging 3.62 ± 0.50 atom% for all days (Table 2, Fig 2). For the FA 18:0, isotopic enrichment averaged 1.87 ± 0.30 atom% between the different batches (Table 2, Fig 2). Enrichment was significantly different between batches (ANOVA, F = 21.66, df = 3,67, *p* = 6*10^−10^), being higher on days 1 and 5 than on day 4 (Tukey, *p* = 0.03) and on day 2 (Tukey, *p* < 1*10^−7^), and higher on day 4 than on day 2 (Tukey, *p* = 5*10^−4^). The major FAs cited above (or PUFAs in general) showed the highest enrichment values within the FAs (Table 2).

**Table 2:** Total fatty acid (TFA) and FA-specific (> 1% TFA) ^13^C isotopic enrichment for *C*. *weissflogii* (atom%) and *L*. *conchilega* (APE); and carbon assimilation (C_assim_, as ng C ind^-1^ (% total C assimilated)) and turnover rate (C_turn_, as % day^-1^) for *L*. *conchilega*.

	*C*. *weissflogii*	*L*. *conchilega*		
FA	atom%	APE	C_assim_	C_turn_
TFA	3.62 ± 0.50	1.16 ± 0.48	199 ± 21	6 ± 1
14:0	3.72 ± 0.53	0.93 ± 0.13	6 ± 1 (3)	5 ± 1
16:0	3.62 ± 0.52	1.21 ± 0.14	44 ± 4 (22)	7 ± 1
16:1(n-7)	3.72 ± 0.54	1.67 ± 0.09	14 ± 1 (7)	9 ± 0
16:1(n-5)	3.48 ± 0.52	N.D.	N.D.	N.D.
16:2(n-4)	3.73 ± 0.52	1.94 ± 0.02	4 ± 0 (2)	11 ± 0
16:3(n-4)	3.73 ± 0.52	2.01 ± 0.06	2 ± 1 (1)	11 ± 0
18:0	1.87 ± 0.30	0.66 ± 0.11	20 ± 2 (10)	7 ± 1
18:1(n-11)	N.D.	0.67 ± 0.10	6 ± 1 (3)	4 ± 1
18:1(n-9)	3.19 ± 0.55	N.D.	N.D.	N.D.
18:1(n-7)	3.26 ± 0.45	0.97 ± 0.11	9 ± 1 (4)	5 ± 1
18:2(n-6)	3.68 ± 0.51	N.D.	N.D.	N.D.
18:4(n-3)	3.76 ± 0.54	1.91 ± 0.09	2 ± 0 (1)	11 ± 0
20:1(n-11)	N.D.	0.49 ± 0.08	2 ± 0 (1)	3 ± 0
20:5(n-3)–EPA	3.74 ± 0.53	1.26 ± 0.12	41 ± 4 (21)	7 ± 1
22:5(n-3)	N.D.	0.94 ± 0.11	25 ± 2 (13)	5 ± 1
22:6(n-3)–DHA	3.76 ± 0.53	1.42 ± 0.10	21 ± 2 (11)	8 ± 1
ΣSFA	3.30 ± 0.90	0.95 ± 0.24	72 ± 7 (36)	7 ± 1
ΣMUFA	3.41 ± 0.51	0.90 ± 0.49	31 ± 3 (15)	5 ± 1
ΣPUFA	3.70 ± 0.45	1.46 ± 0.44	97 ± 11 (49)	7 ± 1

The atom% value for TFA in *C*. *weissflogii* does not include values for the FA 18:0. SFA: saturated FA; MUFA: monounsaturated FA; PUFA: polyunsaturated FA. Values are mean ± SD(n = 3 for larvae (50 individuals pooled per sample) and n = 4 for diatoms). N.D.: not detected.

### Larval composition and enrichment

Approximately 85% of the *L*. *conchilega* larvae survived at the end of the experiment. The average *in situ* DM was 55 μg ind^-1^, and *in situ* C and N contents of larvae were 7668 and 1929 ng ind^-1^, respectively. The average DM, C, and N values at the end of the experiment were 52 μg ind^-1^ and 8511 and 2170 ng ind^-1^, respectively (Table 1). Changes in DM (t = 0.59751, df = 2, *p* = 0.61) and in C (t = -0.42141, df = 2, *p* = 0.71) and N (t = -0.54494, df = 2, *p* = 0.64) contents between larvae sampled *in situ* and after the experiment were not significant, indicating that the organisms did not lose or gain weight during the five-day incubation. The molar C:N ratio of larvae remained the same throughout the experiment at an average of 4.6 (t = -0.02381, df = 2, *p* = 0.98). The average lipid content was similar between *in situ* (632 ng ind^-1^) and experimental (845 ng ind^-1^) individuals (t = -3.4065, df = 2, *p* = 0.076, Table 1). The lipid C content accounted for approximately 7% of the total C content of larvae sampled *in situ* and at the end of the experiment. Total lipid content comprised 1.2 ± 0.3% DM for *in situ* larvae and 1.7 ± 0.4% DM for larvae at the end of the experiment.

**Fig 3:**
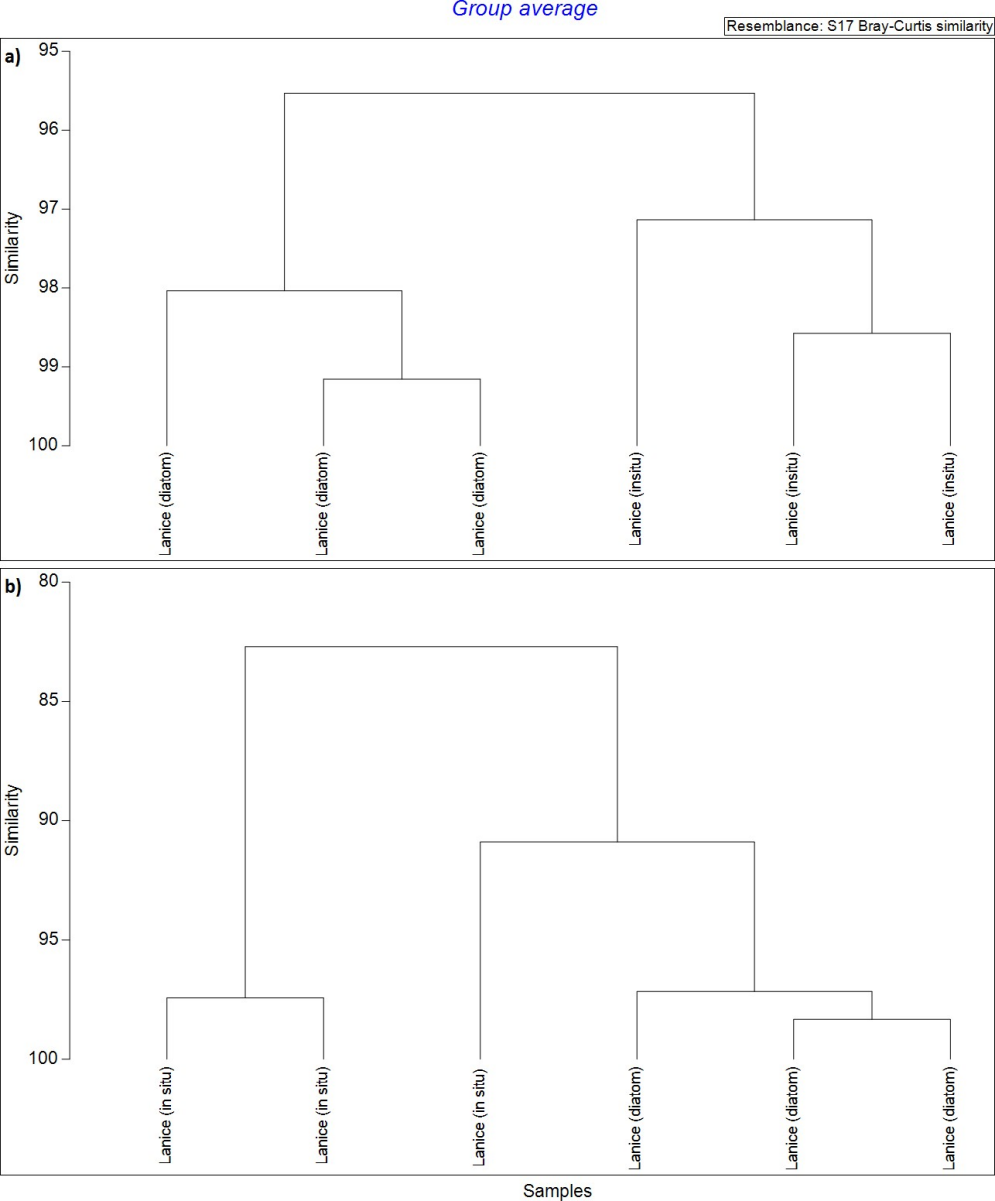
Dendrograms generated with Bray-Curtis similarity matrices comparing fatty acid profiles of *L*. *conchilega* larvae. Similarity matrices present data on the FA profile of larvae sampled *in situ* and after 5 days of feeding on the diatom *C*. *weissflogii*. Only FAs with relative content > 1% TFA were used to generate the similarity matrices. a) logit transformed relative values for FA composition (% TFA). b) absolute (ng C ind^-1^) values for FA composition.

The major FAs in *L*. *conchilega* larvae were EPA, 16:0 and 22:5(n-3), which corresponded to 22, 20, and 15% TFA at the beginning and to 19, 22, and 15% TFA at the end of the experiment, respectively (Table 1). About a quarter of the FAs identified were only present in small amounts (<1% TFA, Fig 1). The relative FA composition of *L*. *conchilega* larvae fed with diatoms remained similar to that of the individuals collected *in situ* (Table 1, Fig 1), as conveyed by the 95.5% similarity between samples shown in the Bray-Curtis dendrogram (Fig 3A). A similar pattern was observed for the absolute FA composition of the larvae (Table 1, Fig 1), except that one of the *in situ* replicates was grouped with the experimental ones at 91% similarity, and these were found to be 82% similar to the other *in situ* replicates (Fig 3B). These results further reinforce the above mentioned statistically non-significant differences in lipid C between *in situ* larvae and those fed during the experiment. In general, changes (i.e., increase or decrease) in the absolute concentration of a FA matched the change in its relative concentration (Fig 1). The only exception to this pattern were the FAs 18:0, 18:1(n-7) and EPA, whose relative concentrations in the larvae decreased despite an increase in their absolute concentrations (Fig 1).

The average ^13^C enrichment of *L*. *conchilega* FAs was of 1.16 ± 0.48 APE, ranging from 0.49 APE in FA 20:1(n-11) to 2.01 APE for FA 16:3(n-4) (Table 2). The FAs 18:1(n-11), 20:1(n-11), and 22:5(n-3) had the lowest enrichment values (together with 18:0) in *L*. *conchilega*, whereas the FAs 16:2(n-4), 16:3(n-4), and 16:1(n-7) (diatom fatty acid trophic markers, FATM) had the highest APE (together with 18:4(n-3)).

### Origin of FAs in the larvae

The enrichment of FAs with ^13^C enabled for the discrimination of the source of each FA-specific lipid C content: internal (Table 1), for FAs already present in the *in situ* larvae, and dietary (Table 2), for FAs assimilated via feeding on the enriched diatoms. The absolute content of internal FAs at the end of the experiment remained the same (~98%) as recorded from *in situ* larvae for two of the three replicates, and on average represented 92% of the *in situ* internal FA content (Table 1). Given the similarity of C content of internal FA between the beginning and end of the experiment the FA-specific differences are not considerable. The internal FA that showed the greatest decrease in terms of C content during the experiment was EPA, which fell from 22% TFA in *in situ* larvae to 19% of the total internal FA in diatom-fed larvae (Table 1). Saturated FAs also showed a slight decrease in C content between the beginning (38% TFA) and end (36% TFA) of the experiment.

### Lipid C assimilation and turnover in larvae

**Fig 4:**
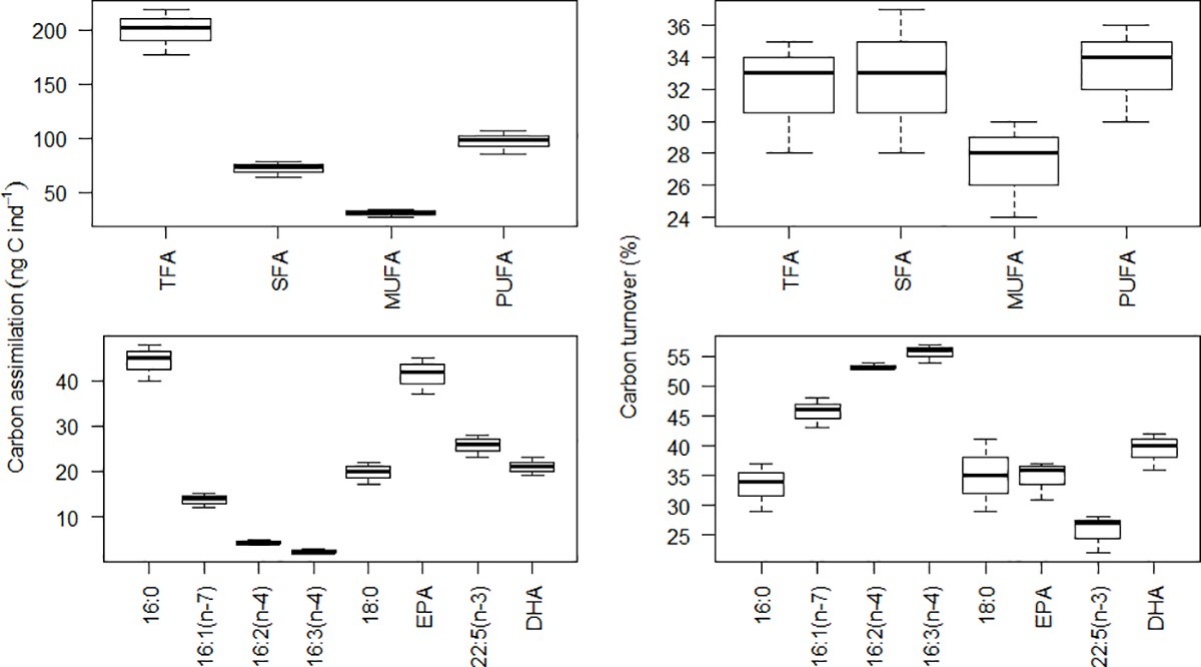
FA-specific carbon assimilation (ng C ind^-1^, left panels) and turnover (%, right panels) in *L*. *conchilega* larvae after the feeding experiment (n = 3). Values are shown for the sum of all fatty acids (TFA), saturated (SFA), monounsaturated (MUFA), and polyunsaturated (PUFA) fatty acids (upper panels); and for specific FAs (lower panels).

Total assimilation of C into lipids in polychaete larvae at the end of the experiment was approximately 200 ng lipid C ind^-1^ (Table 2). The relative assimilation of lipid C into FAs (Table 2) followed the relative composition of internal FAs (Table 1). Approximately half of the lipid C was assimilated into PUFAs, a third into SFAs, and only a sixth into MUFAs (Table 2, Fig 4). The majority of the total lipid C was assimilated into 16:0 and EPA (22 and 21%, respectively), followed by 22:5(n-3), DHA, and 18:0 (13, 11 and 10%, respectively). It is worth noting that the incorporation of dietary C into specific FAs did not follow their relative availability in the diet. The absolute concentration of the FAs 17:0, 18:1(n-11), 20:1(n-11), and 22:5(n-3) increased in *L*. *conchilega*, even though they were absent from the diatom cultures fed to the larvae (Table 2). The absolute concentration of the FAs 18:0 and DHA also increased in the diatom-fed larvae (Table 2), even though they were available in low amounts in the diet (1 and 5% TFA in *C*. *weissflogii*, respectively). Furthermore, the FAs i-15:0, 16:1(n-5), 18:2(n-6), 18:3(n-6), and 20:4(n-3) were only identified in *C*. *weissflogii* (Fig 1) and were either not assimilated or bioconverted by the polychaete larvae. Carbon assimilation in FATM, such as 16:1(n-7), 16:2(n-4), and 16:3(n-4), was low in comparison to that into other FAs, but amounted to the highest C turnover rates (Table 2).

The average lipid C turnover rate was of 6% day^-1^, but varied between 3 and 11% day^-1^ for different FAs (Table 2). Carbon turnover of SFA and PUFA was similar, 7% day^-1^, and slightly higher than that of MUFAs, 5% day^-1^. Overall, 32% of all lipid C was replaced with enriched dietary lipid C by the end of the experiment (Fig 4).

## Discussion

This study is the first to our knowledge to present data on C assimilation and turnover as well as on FA bioconversion in the larvae of the polychaete *L*. *conchilega*, and in any other meroplanktonic larvae for that matter. Although these were recorded from a short-term feeding experiment (5 days), they indicate how quickly polychaete larvae can assimilate dietary material. These lipid-poor larvae seem to be able to regulate their lipid composition (homeostasis) by preferentially assimilating certain FAs and/or by controlling their FA metabolism, and through bioconversion of dietary and internal FAs.

### Lipid composition, homeostasis, and bioconversion in L. conchilega larvae

The low total lipid content recorded for *in situ* larvae in the present study (1.2% DM) is similar to that reported for field and starving crab zoeae and bivalve larvae [39–41]. *Lanice conchilega* larvae thus appear to be lipid-poor organisms.

The relative concentrations of single FAs and of SFAs, MUFAs, and PUFAs were preserved in the larvae during the feeding period. This occurred despite the provision of, and successful feeding upon, a diet with a different FA composition. These results indicate that *L*. *conchilega* larvae can selectively accumulate FAs and/or control its FA metabolism and, thus, regulate lipid composition. A quasi-homeostatic response to variation in FA availability in the food source has also been shown for other zooplankton (daphnids) by Müller-Navarra, 2006 [42]. The preferential retention of a FA over another can be easily identified if their relative proportions in the diet are similar, but their assimilation by the larvae differs. In the present study this is the case for the pairs of FAs 16:0 + 16:1(n-7), EPA + 16:3(n-4), and DHA + 18:4(n-3), for which the first FA of each pair was assimilated by *L*. *conchilega* in quantities that were 3-20x higher than those of the second FA, indicating their preferential accumulation. The FAs EPA and DHA have an important function for marine consumers as they fuel reproductive processes and neural function, and regulate cell membrane fluidity [43–45]. It has been observed that adult *L*. *conchilega* accumulate (n-3) FAs to store energy during gametogenesis [8], but larvae probably use EPA and DHA for growth, as tissue hormones (eicosanoids) are produced from PUFA [46]. The FA 16:0, on the other hand, can be elongated and desaturated into other necessary FAs [23].

There are three different metabolic sources from which the FAs composing the lipid reserves of a consumer can be derived. The FAs can be (1) assimilated unmodified from the food (dietary origin), (2) synthesized *de novo* (dietary or internal origin), or (3) bioconverted (synthesized via chain elongation and desaturation) from another FA of dietary or internal origin [47]. The bioconversion of FAs has more often been reported for crustaceans [23,32] than for polychaetes [48]. In the present study we report the bioconversion of the FAs 17:0, 18:1(n-11), 20:1(n-11), and 22:5(n-3). It should be noted that the synthesis of odd-chained FAs is uncommon in consumers, and that the FA 17:0 is usually considered a bacterial FATM [23]. The presence of this FA in the lipid profile of *L*. *conchilega* larvae indicates that either the organisms are able to synthesize odd-chained FAs or that bacteria were present in small amounts in the experimental units and were ingested by the larvae. Bioconversion occurred for both endogenously-derived FAs, which increased in absolute content during the experiment, and for dietary-derived FAs, which were enriched in ^13^C. The elongation and desaturation of FAs involve reactions catalyzed by enzymes and are subject to isotopic fractionation [49], which could explain why the ^13^C enrichment was lower in the bioconverted FAs. The FA 22:5(n-3) was bioconverted at high quantities and made up 13% of all C assimilated by the larvae. Although marine invertebrates are not able to biosynthesize *de novo* the FAs 18:2(n-6) and 18:3(n-3), they have a limited ability to convert them into PUFAs via chain elongation and desaturation [23,50]. In the present study neither the diet nor the larvae contained 18:3(n-3), so 22:5(n-3) must have been bioconverted from EPA, as has been suggested for brittle stars [51]. It is noteworthy to mention the high enrichment of the FA 18:4(n-3), which is known to be a FATM for dinoflagellates, in the larvae. Although the connection between the referred FA and dinoflagellates might lead to confusion, in the present study the larvae obtained the FA 18:4(n-3) from their diet–this FA was present in the diatom cultures and attained one of the highest atom% values among diatom FAs.

### Lipid homeostasis in lipid-poor and lipid-rich marine planktonic organisms

The ability to regulate FA composition does not come without an energetic cost. Lipid homeostasis can be easy to maintain when an organism feeds upon a prey of similar lipid composition, but will require that energy be allocated to bioconversion if prey have a different biochemical make-up. Furthermore, the way in which an individual utilizes its assimilated FAs is life-stage specific [23]. Based on our results and on the available literature on lipid content and assimilation of mero- and holoplanktonic marine species ([26,27,52,53], other references shown in Table 3, Franco-Santos et al., unpublished data), we put forward the hypothesis that 3 different patterns in lipid homeostasis can be found among lipid-poor and lipid-rich planktonic organisms.

**Table 3:** Total lipid content (TLC, in % dry mass) and wax ester content (WE, in % total lipid content) of several meroplanktonic and holoplanktonic species.

SPECIES	LOCATION	TLC	WE	REFERENCE
**Meroplankton (lipid-poor)**				
**Bivalvia**				
*Crassostrea gigas* larvae	Bay of Archacon, France	2–8	-	[41]
*Teredo navalis* larvae (lab-reared)	Great Harbor, MA, USA	2–4	-	[53]
*Bankia gouldi* larvae (lab-reared)	Pivers Island, NC, USA	3–8	-	[53]
*Crassadoma gigantea* larvae (lab-spawned)	?	4–8	-	[54]
**Bryozoa**				
*Celleporella hyalina* larvae (lab-spawned)	Menai Strait, UK	6–9	-	[55]
**Cirripedia**				
*Balanus balanoides*	Menai Strait, UK	13–15	-	[56]
**Cephalopoda**				
*Octopus vulgaris* hatchlings	Ría de Vigo, Spain	< 15	< 2	[57]
**Decapoda**				
*Campylonotus vagans* zoeae (lab-reared)	Beagle Channel, Argentina	7–9	-	[58]
*Carcinus maenas* zoeae	Helgoland	1–9	-	[40]
*Nephrops norvegicus* larvae	Mediterranean and Irish Seas	6–8	-	[59]
*Panulirus cygnus* larvae	Western Australia	9–13	-	[60]
**Vertebrata**				
*Pleuragramma antarcticum* (larvae)	Antarctic Peninsula	12	4	[61]
*Solea senegalensis* (lab-spawned eggs/larvae)	Bay of Cadiz, Spain	11–12	-	[62]
**Holoplankton (lipid-poor)**				
**Amphipoda**				
*Cyphocaris richardi*	Antarctic Peninsula	21	11	[61]
*Primno abyssalis*	Bute Inlet, Canada	26	12	[63]
*Hyperia galba*	Bute Inlet, Canada	19	8	[63]
*Eusirus propaperdentatus*	Antarctic Peninsula	22	22	[61]
*Parandania boecki*	Antarctic Peninsula	20	33	[61]
*Themisto gaudichaudii*	Antarctic Peninsula	19	9	[61]
**Annelida**				
*Tomopteris septentrionalis*	Bute Inlet, Canada	22	< 0.5	[63]
**Copepoda**				
*Calanus helgolandicus*	?	12	37	[25]
*Euchaeta marina*	Andaman Sea, India	11	-	[64]
*Metridia gerlachei*	Antarctic Peninsula	21	52	[61]
*Rhincalanus gigas*	Antarctic Peninsula	8	< blank	[61]
*Undinula vulgaris*	Andaman Sea, India	9	-	[64]
**Chaetognatha**				
*Eukrohnia hamata*	Arctic	19	12	[65]
*Sagitta enflata*	Andaman Sea, India	8	-	[64]
*Sagitta elegans*	Bute Inlet, Canada	14	6	[63]
*Sagitta gazellae*	Antarctic Peninsula	17	3	[61]
**Coelenterata**				
*Atolla wyvillei*	Antarctic Peninsula	1	48	[61]
*Beroe cucumis*	Bute Inlet, Canada	13	12	[63]
*Diphyes Antarctica*	Antarctic Peninsula	1	16	[61]
*Pleurobrachia pileus*	Bute Inlet, Canada	9	6	[63]
**Decapoda**				
*Acanthephyra sanguinea*	Andaman Sea, India	14	-	[64]
*Alpheus* sp.	Andaman Sea, India	13	-	[64]
*Lucifer hanseni*	Cochin estuary, India	10–16	-	[66]
*Pasiphaea pacifica*	Bute Inlet, Canada	21	4	[63]
**Euphasiacea**				
*Euphausia diomedeae*	Andaman Sea, India	13	-	[64]
*Euphausia pacifica*	Bute Inlet, Canada	19	1	[63]
**Mysidacea**				
*Siriella* sp.	Andaman Sea, India	11	-	[64]
**Ostracoda**				
*Conchoecia elegans*	Bute Inlet, Canada	17	4	[63]
*Cypridina dentata*	Andaman Sea, India	11	-	[64]
**Tunicata**				
*Salpa thompsoni*	Antarctic Peninsula	24	2	[61]
**Holoplankton (lipid-rich)**				
**Amphipoda**				
*Eurythenes gryllus*	Antarctic Peninsula	55	19	[61]
**Copepoda**				
*Calanoides acutus*	Antarctic Peninsula	45	64	[61]
*Calanus hyperboreus*	Arctic	37–74	34–91	[67]
*Calanus finmarchicus*	Norway	31	71	[68]
*Calanus glacialis*	Svalbard, Norway	70	68	[69]
*Calanus plumchrus*	Bute Inlet, Canada	47	86	[63]
*Heterorhabdus tanneri*	Bute Inlet, Canada	43	69	[63]
*Metridia longa*	Arctic	57	76	[65]
*Paraeuchaeta glacialis*	Arctic	43	72	[65]
**Decapoda**				
*Hymenodora glacialis*	Arctic	35–39	44–62	[65]
**Euphasiacea**				
*Euphausia superba* (subadults/adults)	Weddell and Lazarev Seas	7–40	-	[70]
*Thysanoessa macrura*	Antarctic Peninsula	60	50	[61]
**Pteropoda**				
*Clione limacina*	Bute Inlet, Canada and Arctic	19–31	4–12	[63,65]

Lipid-poor meroplanktonic larvae from decapods, bryozoans, and vertebrates, a.o., generally display total lipid contents in the order of 5–15% DM (Table 3). According to the available literature, total lipid content is on the lower side of this range for bivalve larvae (2–8% DM) and on the higher part for barnacle larvae (13–15% DM) and octopus paralarvae (< 15% DM) (Table 3). Both lecithotrophic (which possess and feed on yolk reserves) and planktotrophic (which feed on plankton) larvae rely on obtaining enough energy to sustain metabolic functions and growth into juvenile stages, and cannot dispense energy for storage purposes. Lipid classes which are destined for energy storage, such as wax esters (WE) and triacylglycerols, represent a small proportion of total lipid content in lipid-poor meroplankton (Table 3). These organisms have specific requirements of FAs to sustain their body functions, such as formation of biomembranes, and will thus display lipid homeostasis, even though this may require the allocation of further energy for FA bioconversion. This is the case of the meroplanktonic larvae of *L*. *conchilega*, as discussed in the previous section. It appears, however, that the larvae of hermatypic and soft corals are lipid-rich (total lipid content reported to range between 41–68% DM; [71, 72]). These would be an exception within the lipid-poor meroplankton, and more information is necessary before we can infer on their ability to regulate lipid composition during the larval stage.

Holoplanktonic organisms with intermediate total lipid content (15–25% DM) include small copepods, tunicates, chaetognaths, and ostracods (Table 3). Generally speaking, the lower end of this range is observed in organisms found in tropical regions, whereas the higher end is recorded for individuals from temperate and polar regions (Table 3). The WE content of organisms varies between different groups, and ranges from values as low as 1% total lipid content to as high as 52% total lipid content (Table 3). These holoplanktonic organisms generally have a small to modest ability for energy storage as lipid reserves, a strategy whose focus is likely the maximization of reproductive output. Such species will mostly assimilate dietary FAs in an unmodified manner (non-homeostatic).

Lipid-rich holoplanktonic species, on the other hand, will display total lipid contents > 30% DM (Table 3). The species for which data were available were mostly from polar regions, and represent amphipods (55% DM), large copepods (31–74% DM), krill (7–60% DM), decapods (35–39% DM), and pteropods (19–31% DM) (Table 3). These organisms need to store energy in the most dense and effective way to survive periods of food shortage during winter and for buoyancy purposes [73], and will thus bioconvert dietary FAs into long chain MUFAs / WE (semi-homeostatic). This can be exemplified by the large proportion of WE within the total lipid content in the mentioned groups, which is approximately 50% in decapods and krill and 34–90% in copepods (Table 3). Only pteropods and amphipods have a lower proportion of WE, which is approximately 15–20% of the total lipid content (Table 3).

### The applicability of the FATM concept

In theory, FATM are assimilated by consumers in a conservative manner [24,25]. The applicability of the FATM concept has been broadly acknowledged to depend, a.o., on the qualitative and quantitative transfer of FAs between trophic levels and on their metabolic stability and non-selective incorporation into consumer tissues [23]. In the present study the diatom FATMs were transferred between the producer and consumer trophic levels in a qualitative manner. In quantitative terms, however, the dietary FAs 16:0, EPA, and DHA were preferentially assimilated by *L*. *conchilega* larvae. Furthermore, we have postulated that EPA, which is a diatom FATM, was likely bioconverted (via chain elongation, as indicated in Fig 1 of [74]) into 22:5(n-3) by the polychaete larvae. Unlike the trends shown by lipid-rich or lipid-accumulating organisms, the results of this study indicate that the applicability of the concept of FATM is limited in the lipid-poor larvae of *L*. *conchilega*. We also suggest that this will be the case for other lipid-poor meroplanktonic larvae, as they probably also display preferential assimilation and bioconversion of FAs in order to sustain body functions.

### Experimental conditions

The active uptake of dietary C during the experiment was sufficient to compensate for the larval metabolic costs of living during the 5 days. This indicates that diatoms are a nutritious source of food for *L*. *conchilega* larvae, which readily accept these algae and utilize it as metabolically necessary. The lack of literature on feeding studies with meroplanktonic larvae prompted us to look for guidelines on food concentrations from laboratory experiments with small planktonic copepods (e.g., [75]), so it is possible that individuals would have shown higher growth if they had received more food.

A common value of 30–40% DM is usually attributed for C content in marine zooplankton (e.g., [76]). The values obtained in the present study for *L*. *conchilega* larvae (~15% DM) were only a third to half of that, but within the 16–44% DM range recorded for polychaete by Parsons et al. [76]. It should be noted, however, that sample C and N contents and weight might have been affected by sampling larvae while they were still inside their tubes. The larval tube is composed of a thin organic layer, to which sand and shell fragments are attached at later stages [21]. An attempt was made to remove them, but we observed that it was not possible to do so without damaging the individuals or without the application of a sedative.

## Conclusions

The present study documented preferential assimilation of FAs by *L*. *conchilega* larvae, which seem to be able to regulate their FA composition regardless of the FA profile of their food source. Bioconversion was also recorded in these individuals, which synthesized the PUFA 22:5(n-3) from dietary FA precursors. Our results show that dietary FAs are not transferred in a conservative (quantitative) manner, indicating that the concept of FATM is of limited applicability in the trophic study of lipid-poor *L*. *conchilega* larvae. This is probably also the case for other lipid-poor meroplanktonic larvae. Based on the results of this study and on data available from the literature, we propose that lipid homeostasis depends upon the lipid content of zooplanktonic organisms. Lipid-poor meroplanktonic larvae should display lipid homeostasis in order to maintain vital lipid-based body functions; holoplanktonic organisms with intermediate lipid levels will usually assimilate dietary lipids in a conservative manner and invest assimilated energy into reproduction; whereas lipid-rich holoplanktonic organisms will be semi-homeostatic, converting stored energy (FAs) into WE in order to survive starvation conditions during winter and to increase buoyancy.
